# Obesity, High Blood Pressure and Monocytosis in Truck Drivers of the Western Border of a State in Southern Brazil: A Cross-Sectional Study

**DOI:** 10.3390/diseases13100314

**Published:** 2025-09-24

**Authors:** Carolina Pereira de Oliveira, Laura Smolski dos Santos, Gênifer Erminda Schreiner, Camila Berny Pereira, Silvia Muller de Moura Sarmento, Itamar Luís Gonçalves, Vanusa Manfredini

**Affiliations:** 1 Undergraduate Nursing Program, Federal University of Pampa, Uruguaiana 97501-970, Rio Grande do Sul, Brazil; 2 Postgraduate Program in Biochemistry, Federal University of Pampa, Uruguaiana 97501-970, Rio Grande do Sul, Brazil; laurasantos.aluno@unipampa.edu.br (L.S.d.S.); geniferschreiner.aluno@unipampa.edu.br (G.E.S.); 3 Undergraduate Pharmacy Program, Federal University of Pampa, Uruguaiana 97501-970, Rio Grande do Sul, Brazil; camilaberny.aluno@unipampa.edu.br; 4 Uruguaiana Municipal Health Department, Border Laboratory, Uruguaiana 97501-653, Rio Grande do Sul, Brazil; mullermoura@gmail.com; 5 Undergraduate Pharmacy Program, Integrated Regional University of Upper Uruguay and Missions, Erechim 99709-910, Rio Grande do Sul, Brazil; itamar3141@yahoo.com.br

**Keywords:** truck drivers, occupational health, obesity, chronic diseases, oxidative stress

## Abstract

Objective: This study aimed to analyze the epidemiological, hematological, and oxidative stress profile of truck drivers. Method: It involved 63 drivers from the western border of a state in southern Brazil who completed a questionnaire, had vital signs and anthropometric evaluations, and provided blood samples. Hematological parameters, leukocytes, and oxidative damage to proteins and lipids were analyzed. Results: A high prevalence of overweight and obesity was found among the drivers, with an increased risk of cardiovascular issues and hypertension. Obese drivers had higher monocyte counts, while those with normal weight had increased protein carbonylation levels. Conclusions: It is crucial to implement health interventions to prevent chronic diseases in truck drivers, given their high exposure to risk factors.

## 1. Introduction

Truck drivers represent a crucial working class for the Brazilian economy, as road transport accounts for 60% of the country’s cargo transport [1]. However, it is still a professional category exposed to precarious and exhausting working conditions, which leads to the adoption of a harmful lifestyle with detrimental habits for their health [2], such as consuming poor-quality food, sedentarism and smoking [3].

Furthermore, it is known that diet and a sedentary lifestyle play a major role in cardiovascular risk and the development of metabolic syndrome [4]. Additionally, they can lead to consequences such as overweight, obesity [5], sleep disorders, stress, and fatigue [1]. Obesity, hypertension, tumors, diabetes mellitus, chronic respiratory diseases, and other cardiovascular conditions are included in non-communicable diseases (NCDs) [6], the leading cause of death globally, constituting a significant challenge for public health [7].

NCDs induced by age and lifestyle have a strong correlation with low-grade chronic inflammation, a condition in which the body continuously experiences high levels of pro-inflammatory cytokines [8]. This inflammation is caused by the production of reactive oxygen species (ROS) resulting from the imbalance between oxidant and antioxidant substances, leading to oxidative stress [9].

In the context of obesity, adipocytes undergo hypertrophy, which subsequently leads to an increased recruitment of immune cells, such as M1 macrophages, and inflammatory mediators within the tissue, including tumor necrosis factor alpha (TNF-α), interleukin 6 (IL-6), and interleukin 1β (IL-1β) [10].

The dry port located in Uruguaiana, in Rio Grande do Sul, Brazil, is the main entry and exit point for road freight transportation due to the border with Argentina [11]. Approximately 13,000 trucks pass through it monthly, with the capacity to accommodate between 16,000 and 17,000 trucks per month [12].

Furthermore, truck drivers represent a population that has been scarcely explored in the scientific literature and remains largely underserved in terms of healthcare. The occupational demands, prolonged sedentary behavior, irregular schedules, and limited access to healthy food options place this group at an increased risk for obesity, hypertension, and other chronic health conditions. Despite these vulnerabilities, few studies have systematically assessed the prevalence of these conditions or evaluated the associated risk factors among truck drivers.

In light of the growing evidence linking lifestyle factors, occupational demands, and adverse health outcomes among truck drivers, further investigation into their biological and clinical profiles is warranted. Therefore, this study aimed to analyze the epidemiological, hematological, and oxidative stress profiles of truck drivers, providing insights into the potential health risks faced by this population and contributing to the development of preventive strategies tailored to their specific occupational context.

## 2. Methods

### 2.1. Ethical Aspects

The present study was approved by the Research Ethics Committee of the Universidade Federal do Pampa (UNIPAMPA), under approval number 5.854.845. The participants of the study signed an informed consent form (ICF) and were invited to complete a sociodemographic and health questionnaire developed by the author.

### 2.2. Study Population

The city of Uruguaiana, Brazil, borders Argentina, and the sample size was calculated considering an average of 75 truck drivers passing through the city, a 95% confidence level, and a sampling error of 5%. Thus, we obtained 63 participants in this study. The sample size calculation was performed according to Krejcie and Morgan (1970) [13].

The study was conducted from February to March 2024, when a group invitation was extended to the participants, and those who consented to participate in the research had their blood pressure measured, with measurements of weight, height, abdominal circumference (AC), and neck circumference (NC), followed by the completion of a questionnaire. After, they were conducted to the nurse’s office and a blood sample was collected. The participants were divided into 3 groups based on their body mass index (BMI) classification: normal weight (18.5–24.9 kg/m^2^), overweight (25–29.9 kg/m^2^), and obesity (≥30 kg/m^2^).

### 2.3. Sample Obtaining and Preparing

Venous blood samples were collected in the morning by a trained professional, with an approximate volume of 10 mL. Samples were placed into two tubes: one containing ethylenediaminetetraacetic acid (EDTA) for whole blood and plasma collection and another with a clot activator gel for serum collection.

All materials were properly refrigerated in a cooler and transported under appropriate conditions to the Hematology and Clinical Cytology Laboratory (Laboratory 411) at the Universidade Federal do Pampa (UNIPAMPA). Once in the laboratory, the whole blood was used to prepare slides by blood smear, and the samples were protected from light, processed, and centrifuged, with aliquots of plasma and serum from each participant being fractionated and stored for subsequent analyses.

### 2.4. Hematological and Oxidative Stress Parameters

For the analysis of the participants’ hematological differential, the slides previously prepared by blood smear were used after being stained with Instant Prov panotic stain from New Prov^®^ (Pinhais, PR, Brazil). Leukocyte counting was performed under an Olympus^®^ optical microscope (Olympus^®^, Hachioji, Tokyo, Japan) with an immersion objective (1000×), and the results were expressed as percentages.

To evaluate the levels of oxidative damage to biomolecules, plasma samples from the participants were used. For the analysis of oxidative damage to proteins, the protein carbonylation method described by Levine et al. [14] was employed. The readings were performed using the Spectramax M5^®^ equipment (Molecular Devices^®^, Sunnyvale, CA, USA) at 370 nm, and the data were expressed as nmol carbonyl/mg protein. For the assessment of oxidative damage to lipids, the thiobarbituric acid reactive substances (TBARS) method described by Ohkawa et al. [15] was used. The readings were taken with the Biospectro SP-22 spectrophotometer (Biospectro, Shimadzu^®^, Kyoto, Japan) at 532 nm, and the results were expressed as nmol malondialdehyde (MDA)/mL.

### 2.5. Statistical Analysis

The results were expressed as mean ± standard deviation. The normality of the data was assessed using the Shapiro–Wilk normality test. For parametric data, one-way Analysis of Variance (ANOVA) was used, followed by the Bonferroni post hoc test, while for non-parametric data, the Kruskal–Wallis test was applied, followed by Dunn’s post hoc test. The software GraphPad Prism 9.0 (San Diego, CA, USA) was used, and data were considered statistically significant at *p* < 0.05.

## 3. Results

### 3.1. Sociodemographic Profile of the Studied Population

The study included 63 men: 4 (6%) normal-weight, 20 (32%) overweight, and 39 (62%) obese. The small size of the normal-weight group limits statistical power and generalizability. The normal-weight participants were also slightly younger (42 ± 14 years) than the overweight (49 ± 13 years) and obesity (50 ± 10 years) groups (Table 1).

The participants were predominantly white in all three groups, with only 5% of the overweight group and 3% of the obesity group self-identifying as Black. Regarding marital status, 70% of the men in the obesity group were married, 53% in the overweight group, and 25% in the normal weight group.

The average salary of truck drivers, in terms of the minimum wage equivalent (MW) to BRL 1412, according to Decree 11.864/23, was 4.9 MW in the normal weight group and 3.7 MW in the obesity group. Regarding education, 46% (*n* = 29) of the participants reported having completed high school, while 29% (*n* = 18) indicated having incomplete elementary education.

### 3.2. Health Status

Among the anthropometric measurements, shown in Table 2, the mean ± standard deviation of NC (cm) stood out in all three groups. Similarly, the mean ± standard deviation of AC (cm) was highlighted for the overweight (102.6 ± 8.4 cm) and obesity (118.8 ± 11.7 cm) groups.

Regarding the chronic diseases reported by the drivers, there was a high prevalence (62%) of obesity among the study participants. Regarding self-reported diseases, there was a prevalence of 23.80% (*n* = 15) of hypertension, mentioned by one driver in the normal weight group, three in the overweight group, and eleven in the obesity group. Similarly, there was a prevalence of 16% (*n* = 10) of diabetes mellitus, reported by three participants in the overweight group and seven in the obesity group.

Among the medications used continuously, the use of antihypertensive drugs, such as potassium losartan, was highlighted, being mentioned eight times (48%) by drivers in the obesity group, and once by truck drivers in the normal BMI (25%) and overweight (17%) groups. Additionally, there was a higher use of continuous medications in the obesity group compared to the other groups.

### 3.3. Lifestyle

Regarding alcohol consumption (Table 3), 62% (*n* = 39) of the participants reported using alcohol. Among them, 10% (*n* = 4) stated that they consume some type of alcoholic beverage every day. On the other hand, 35% of the participants indicated that they do not drink alcoholic beverages.

Concerning tobacco use, 25% (*n* = 1) of the participants in the overweight group and 10% (*n* = 4) in the obesity group reported smoking, in contrast to 76% (*n* = 48) of all the participants who reported not being smokers.

In relation to physical activity, 59% (*n* = 37) of the professionals reported not engaging in any physical exercise, with the obesity group standing out, where 69% (*n* = 27) stated that they do not exercise.

### 3.4. Oxidative Stress Parameters

**Figure 1:**
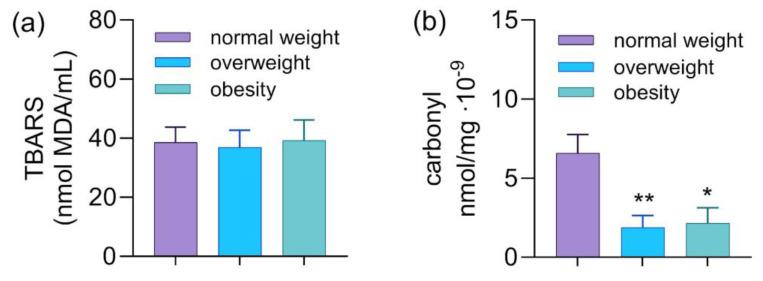
TBARS and protein carbonylation levels in truck drivers. (**a**) Representation of TBARS levels (nmol MDA/mL) in the groups. (**b**) Representation of protein carbonylation levels (nmol carbonyl/mg of protein) in the groups. The * denotes a significant difference compared to the groups being compared (* *p* < 0.05, ** *p* < 0.01) according to the Kruskal–Wallis test followed by Dunn’s post hoc test. Source: developed by the author.

In terms of lipid oxidation (Figure 1), lipid damage was quantified using the thiobarbituric acid reactive substances (TBARS) method, while protein oxidation was measured using the protein carbonylation method, both being spectrophotometric methods. No statistically significant difference was found in TBARS levels. In the quantification of protein carbonylation, a statistically significant increase was observed between the normal weight group (6.6 ± 1.2 nmol/mg·10^−9^) and the overweight group (1.9 ± 0.8 nmol/mg·10^−9^), as well as between the normal weight and obesity groups (2.2 ± 1 nmol/mg·10^−9^).

### 3.5. Peripheral Blood Leukocyte Differential

In the blood cell differentiation (Table 4), a statistically significant increase in the number of monocytes was observed in the obesity group (9 ± 4%) compared to the normal group (3 ± 1%), with no significant difference observed between the other leukocytes.

## 4. Discussion

As demonstrated by Bezerra et al. [16], the Brazilian population has experienced a shift in their eating habits, consuming more products such as chicken, eggs, and ultraprocessed foods while, on the other hand, having fewer traditional meals of the country. Consequently, Malta et al. [17], along with Bezerra et al. [16], revealed an increase in the prevalence of overweight and obesity in the Brazilian population.

Among the truck drivers interviewed, there was a high prevalence (62%) of obesity and a low number of normal-weight drivers (6%). The study by Cavagioni et al. [18], involving 258 men conducted in the state of São Paulo, showed that 45% of workers were overweight and 37% were obese, a figure lower than that found in the present investigation. A study conducted with urban bus drivers in the United States found a prevalence of 59% of obese participants, showing a result similar to that found in our study [19].

Regarding the low number of participants with normal weight, it is believed that this issue may be attributed to the lifestyle habits of the professionals, characterized by a high intake of readily available ultra-processed foods, physical inactivity, smoking, alcohol consumption, and the absence of a consistent daily routine due to the nature of their work [20].

It was also found that these professionals, in addition to having a sedentary occupation, do not usually engage in physical exercise (59%). The study conducted by Codarin et al. [21] found an association between physical activity, higher levels of education, and lower alcohol consumption, corroborating the findings of the present study, where 69% of the participants who exercised (*n* = 26) had completed high school. However, a higher alcohol consumption (72%) was found among participants with a high school education compared to those with incomplete elementary education (61%). Another investigation showed that these professionals tend to engage little in physical activities and overestimate their level of activity in their reports [22].

According to Hu et al. [23] and Silva et al. [24], AC and NC measurements are good indicators of obesity and cardiometabolic risk. The study by Pimenta et al. [25] suggests that when AC values exceed 102 cm in men, there is a high risk of developing hypertension, dyslipidemia, and diabetes mellitus. Furthermore, Pimenta’s study [25] highlights that a NC greater than 37 cm in males is a risk marker for cardiovascular disorders, especially hypertension.

Regarding NC, it was found that no driver had a measurement below 37 cm, while two (3%) showed a borderline measurement. Thus, the data found corroborate these findings, as 40% (*n* = 19) of the participants with AC > 102 cm (*n* = 47) and 38% (*n* = 22) of those with NC > 37 cm (*n* = 58) reported having developed some chronic health complication.

Hypertension is influenced by both genetic predisposition and environmental factors. While rare single-gene forms exist, most cases result from multiple small-effect genetic variants. Lifestyle factors, such as obesity, high sodium intake, low potassium intake, physical inactivity, and alcohol consumption, play a major role in elevating blood pressure, whereas regular exercise and a balanced diet can mitigate risk. Early-life factors, including childhood blood pressure, prematurity, and low birth weight, also contribute to future hypertension [26].

According to the guidelines from the American College of Cardiology/American Heart Association [26], hypertension is diagnosed when blood pressure exceeds 130/80 mmHg. Meanwhile, the Brazilian Guidelines [27] considers values between 130/80 mmHg and 139/89 mmHg as pre-hypertension, with hypertension starting from 140/90 mmHg. All organizations warn about the risks of values exceeding 130 mmHg for systolic blood pressure and 85 mmHg for diastolic blood pressure. Within the truck drivers, 11% (*n* = 7) had an systolic blood pressure below 120 mmHg, considered optimal; of these, six belonged to the obesity group.

Meanwhile, 19% (*n* = 12) showed values consistent with hypertension (≥140/90 mmHg), of whom five had a previous diagnosis of hypertension and eight belonged to the obesity group, while 58% (*n* = 7) reported not having hypertension and not using any medication for blood pressure control. The low medication usage rate recorded may be due to limited access to healthcare services due to the lack of a fixed routine [28]. Even so, antihypertensive medications (69%) were the most commonly used among the 13 drugs mentioned by the professionals.

NCDs are the leading causes of death worldwide [7] and, in addition, they have a significant impact on health, social security, and the economy of countries [3]. The most common chronic diseases are hypertension, diabetes mellitus, and dyslipidemia [29]. Among the 63 truck drivers, a prevalence of 24% (*n* = 15) for hypertension, 16% (*n* = 10) for diabetes mellitus, and 5% (*n* = 3) for dyslipidemia was found. The results obtained in this study, except for the alteration in blood lipids, were higher than those reported by Girotto et al. [28], who found that, among 683 truck drivers, 16% reported having hypertension, 13% had dyslipidemia, and 8% had diabetes mellitus.

Regarding lifestyle habits, the use of alcohol and other drugs is a factor that increases the risk of road accidents [30] and is a major public health issue due to the consequences it brings to the body [31]. In the present study, there was a high prevalence (62%) of alcohol consumption among the drivers, and, as mentioned by García-Perales [31], these results may be attributed to long working hours, sleep deprivation, and being away from family.

Alcohol also exerts an effect on plasma levels of vitamins and antioxidants, which contributes to oxidative stress [32]. Similarly, other lifestyle habits are among the exogenous sources of oxidative damage, such as tobacco and drug use, diet, exposure to radiation, and pollution [33].

The accumulation of fat and excessive calorie consumption increase oxidative damage to cells [34], activating the inflammatory process and the secretion of pro-inflammatory cytokines [9]. Oliveira et al. [35] state that obesity is a chronic inflammation, causing damage through pro-inflammatory substances and promoting the infiltration of macrophages into adipose tissue.

Normal adult monocyte counts range from 0.2–0.8 × 10^9^/L, generally slightly higher in males and minimally influenced by race, with monocytes being more responsive to inflammatory stimuli in men, likely due to sex hormones. Persistent monocytosis is defined by the World Health Organization (WHO) as ≥1 × 10^9^/L and ≥10% of leukocytes for over 3 months [36]. Monocyte levels, which are affected by body fat and associated with conditions such as coronary artery disease [35], pre-diabetes, and diabetes mellitus [37], were significantly higher in the obese group in the present study (9 ± 4 %) compared to the normal-weight group (3 ± 1 %), consistent with findings in adolescents reported by Tenório et al. [38]. Although monocyte levels among obese truck drivers were within the normal range, the values observed remain close to the clinical threshold for monocytosis.

Lipid peroxidation, measured through TBARS, has been strongly associated with cardiac events, independent of other comorbidities [39]. Similarly, elevated protein carbonylation indicates severe oxidative damage and protein dysfunction caused by diseases, while moderate carbonylation can activate or inhibit the functions of target proteins, as well as promote their selective degradation by the proteasome [40].

Horn et al. [41] reported higher lipoperoxidation and protein carbonylation in obese patients compared to controls, attributing this to mechanisms such as pro-inflammatory cytokine release from adipose tissue, altered mitochondrial metabolism, hyperleptinemia, and excessive nutrient intake. Collectively, these factors promote ROS overproduction, favoring lipid peroxidation, protein carbonylation, and consequent cellular oxidative damage. However, in the present study, no similar pattern was observed, as protein carbonylation levels were unexpectedly higher in the normal-weight group. This finding may be explained by potential confounding factors, such as the use of specific medications or the presence of underlying health conditions capable of promoting oxidative damage independently of adiposity. Lifestyle-related aspects, including alcohol intake, smoking, or dietary habits, may also have contributed to these results.

A limitation of the present study is that the sample of truck drivers may not fully represent the broader population in southern Brazil. Variations in age, route length, and duration of employment could affect the generalizability of the findings, while the very small number of normal-weight participants reduces statistical power and may have influenced the interpretation of protein carbonylation results.

Furthermore, a longitudinal design would be required to determine whether elevated monocyte counts in this population progress to persistent monocytosis, as defined by WHO criteria. Future studies with larger and more diverse samples are needed to confirm whether the observed results are applicable to the wider truck driver population in the region. Although sociodemographic and health-related characteristics were assessed, the lack of an in-depth analysis of these variables may have limited the identification of potential confounders.

## 5. Conclusions

The study revealed that truck drivers face high rates of obesity and other chronic non-communicable diseases, such as hypertension, diabetes mellitus, and dyslipidemia, as well as a high number of cases of untreated hypertension and alcohol consumption. A significant increase in monocytes was observed in obese drivers, while elevated protein carbonylation levels were found in drivers with normal weight, highlighting the complex interplay between metabolic and oxidative stress markers in this population.

By focusing on this population, the present study provides valuable insights into the health status of a group that is both at high risk and underrepresented in research. The findings contribute to filling existing knowledge gaps and help inform public health strategies aimed at improving the prevention, early detection, and management of chronic diseases within this occupational group.

Given the high risk of chronic diseases among truck drivers, establishing specialized occupational health units in strategic locations, such as high-traffic areas and sites where drivers wait for cargo release, would play a key role in reporting work-related health issues while also providing preventive care and health promotion, including screenings, risk management guidance, and lifestyle education, in order to enhance early detection and help reduce the burden of chronic conditions in this high-risk population.

Also, educational campaigns on hypertension, nutrition, and physical activity could be implemented at company bases and rest stops. Structured opportunities for exercise during mandatory breaks, along with partnerships with roadside restaurants to provide healthier meals, may further promote well-being. In addition, workplace policies encouraging healthy lifestyle behaviors, such as scheduled breaks, access to healthy food options, and ergonomic adjustments in vehicles may help mitigate the adverse health effects associated with long hours of driving and sedentary work.

In the Brazilian context, feasible interventions to improve the health of truck drivers including the use and dissemination of the Cartão do Caminhoneiro (Truck Driver Health Card) could be intensified, allowing drivers to record and track their clinical information, monitor blood pressure, weight, medications, and vaccinations, and receive guidance on healthy lifestyle practices. Finally, the development of a national integrated health information system would allow healthcare professionals across the country to access drivers’ medical records and clinical data in real time, ensuring continuity of care and facilitating timely interventions regardless of the driver’s location.

Although the Programa de Controle Médico de Saúde Ocupacional (Occupational Health Medical Control Program, PCMSO), as established by the Brazilian Regulatory Standard No. 7 (NR-7), already provides a framework for regular medical surveillance of workers, a key challenge lies in ensuring effective enforcement and adherence. In the case of truck drivers, a high-risk occupational group, improvements should prioritize strengthening monitoring and inspection mechanisms to verify whether companies are complying with legal requirements, such as periodic consultations, laboratory tests, and toxicological examinations. Additionally, greater efforts are needed to guarantee worker participation and to integrate health information into centralized systems, thereby ensuring continuity of care across regions. Rather than creating new regulations, enhancing compliance with existing policies could significantly improve occupational health outcomes for truck drivers.

## Figures and Tables

**Table 1:** Truck drivers sociodemographic data. Descriptive analysis. Source: developed by the authors. Data presented as absolute frequencies, relative frequencies, and mean ± standard deviation. Legend: *n*—absolute frequency. %—relative frequency.

	GROUPS		
Parameters	Normal (*n* = 4)	Overweight (*n* = 20)	Obesity (*n* = 39)
Age	42 ± 14	49 ± 13	50 ± 10
Marital status			
Single	2 (75%)	6 (32%)	8 (21%)
Stable union	-	1 (5%)	3 (8%)
Married	1 (25%)	10 (53%)	27 (69%)
Divorced	-	1 (5%)	1 (3%)
Widower	-	1 (5%)	-
Color			
White	2 (75%)	10 (50%)	23 (59%)
Mixed race	1 (25%)	9 (45%)	15 (39%)
Black	-	-	1 (3%)
Income (MW)	4.9 ± 3.1	4.2 ± 3.3	3.7 ± 1.3
Education			
Incomplete elementary education	-	4 (20%)	14 (36%)
Complete elementary education	2 (25%)	3 (15%)	7 (18%)
Incomplete high school	-	1 (5%)	2 (5%)
Completed high school	3 (75%)	10 (50%)	16 (41%)
Incomplete higher education	-	1 (5%)	-
Completed higher education	-	1 (5%)	-

**Table 2:** Anthropometrics and health data. Source: developed by the authors.

	GROUPS		
Parameters	Normal (*n* = 4)	Overweight (*n* = 20)	Obesity (*n* = 39)
Weight (Kg)	76.3 ± 6.1	84.7 ± 8.4	105 ± 13.1
Height (m)	1.8 ± 0.1	1.7 ± 0.1	1.7 ± 0.1
BMI (Kg/m^2^)	24.2 ± 0.4	27.7 ± 1.6	34.4 ± 3.4
Abdominal circumference (cm)	92.3 ± 6.0	102.6 ± 8.4	118.8 ± 11.7
Neck circumference (cm)	39.5 ± 1.9	42.3 ± 3.4	45.3 ± 2.8
Systolic blood pressure (mm/Hg)	123.3 ± 5.8	135.4 ± 20.3	134.6 ± 21.0
Diastolic blood pressure (mm/Hg)	83.3 ± 5.8	90.8 ± 13.8	89.2 ± 12.9
Self-reported chronic diseases			
Hypertension	1 (25%)	3 (30%)	11 (57.9%)
Diabetes mellitus	-	3 (30%)	7 (36.8%)
Dyslipidemias	-	2 (20%)	1 (5.3%)
Liver cirrhosis	-	1 (10%)	-
Cancer	-	1 (10%)	-
Medications for continuous use			
Potassium losartan	1 (25%)	1 (17%)	8 (42%)
Atenolol	1 (25%)	-	1 (5%)
Allopurinol	-	1 (17%)	-
Metformin hydrochloride	-	1 (17%)	2 (11%)
Ciprofibrate	-	1 (17%)	-
Amlodipine besilate	-	1 (17%)	-
Losartan + Amlodipine	-	1 (17%)	-
Indapamide	-	-	1 (5%)
Omeprazole	-	-	1 (5%)
Clonazepam	-	-	1 (5%)
Hydrochlorothiazide	-	-	1 (5%)
Enalapril maleate	-	-	2 (11%)
Valsartan	-	-	2 (11%)

Data presented as absolute frequencies, relative frequencies, and mean ± standard deviation. Legend: *n*—absolute frequency. %—relative frequency.

**Table 3:** Alcohol consumption, tobacco use, and physical activity. Source: developed by the author.

	GROUPS		
Parameters	Normal (*n* = 4)	Overweight (*n* = 20)	Obesity (*n* = 39)
Alcohol consumption			
Yes	2 (50%)	12 (63%)	25 (66%)
Yes, rarely	-	1 (5%)	3 (8%)
Yes, once a week	2 (50%)	8 (42%)	18 (47%)
Yes, two to four times a week	-	2 (11%)	1 (3%)
Yes, everyday	-	1 (5%)	3 (8%)
No	2 (50%)	7 (37%)	13 (34%)
Tobacco consumption			
Yes	1 (25%)	5 (25%)	4 (11%)
No	2 (75%)	13 (72%)	33 (89%)
Physical activity practice			
Yes	3 (75%)	11 (55%)	12 (31%)
No	1 (25%)	9 (45%)	27 (69%)

Data presented as absolute frequencies and relative frequencies. Legend: *n*—absolute frequency. %—relative frequency.

**Table 4:** Leukocyte differential count. Source: developed by the author.

	Groups		
Parameters	Normal	Overweight	Obesity
Neutrophils (%)	57.8 (±4.3)	57.2 (±8)	54 (±10.4)
Lymphocytes (%)	32 (±7)	32.6 (±9.5)	34.1 (±9.7)
Monocytes (%)	3 (±1)	7.8 (±4.3)	9 (±4) *
Eosinophils (%)	2.8 (±2.5)	3.1 (±2.5)	2.8 (±2.5)
Basophils (%)	0.3 (±0.5)	0.1 (±0.2)	0.1 (±0.0)
Band cells (%)	0.0	0.3 (±0.1)	0.3 (±0.1)

Data presented as mean and standard deviation (in %). The * denotes a significant difference between the obesity group and the normal weight group (* *p* < 0.05) according to the Kruskal–Wallis test followed by Dunn’s post hoc test.

## Data Availability

The data presented in this study are available on request from the corresponding authors. The data are not publicly available due to privacy and ethical restrictions.
